# Was Mandatory Quarantine Necessary in China for Controlling the 2009 H1N1 Pandemic?

**DOI:** 10.3390/ijerph10104690

**Published:** 2013-09-30

**Authors:** Xinhai Li, Wenjun Geng, Huidong Tian, Dejian Lai

**Affiliations:** 1 Key Laboratory of the Zoological Systematics and Evolution, Institute of Zoology, Chinese Academy of Sciences, 1-5 Beichen West Road, Chaoyang District, Beijing 100101, China; E-Mail: tienhuitung@gmail.com; 2 Chia Tai Tianqing Pharmaceutical Group Co., Ltd., 9 Huiou Road, Nanjing Economic Development Zone, Nanjing 210038, China; E-Mail: wintergeng@gmail.com; 3 School of Public Health, University of Texas, 1200 Herman Pressler Street, Suite 1006 Houston, TX 77030, USA; E-Mail: dejian.lai@uth.tmc.edu; 4 Faculty of Statistics, Jiangxi University of Finance and Economics, Nanchang 330013, China

**Keywords:** China, 2009 H1N1 pandemic, prevention policy, quarantine

## Abstract

The Chinese government enforced mandatory quarantine for 60 days (from 10 May to 8 July 2009) as a preventative strategy to control the spread of the 2009 H1N1 pandemic. Such a prevention strategy was stricter than other non-pharmaceutical interventions that were carried out in many other countries. We evaluated the effectiveness of the mandatory quarantine and provide suggestions for interventions against possible future influenza pandemics. We selected one city, Beijing, as the analysis target. We reviewed the epidemiologic dynamics of the 2009 H1N1 pandemic and the implementation of quarantine measures in Beijing. The infectious population was simulated under two scenarios (quarantined and not quarantined) using a deterministic Susceptible-Exposed-Infectious-Recovered (SEIR) model. The basic reproduction number *R_0_* was adjusted to match the epidemic wave in Beijing. We found that mandatory quarantine served to postpone the spread of the 2009 H1N1 pandemic in Beijing by one and a half months. If mandatory quarantine was not enforced in Beijing, the infectious population could have reached 1,553 by 21 October, *i.e*., 5.6 times higher than the observed number. When the cost of quarantine is taken into account, mandatory quarantine was not an economically effective intervention approach against the 2009 H1N1 pandemic. We suggest adopting mitigation methods for an influenza pandemic with low mortality and morbidity.

## 1. Introduction

Influenza, a common infectious disease in humans, recurs annually and has been modeled more thoroughly than any other infectious disease [1]. Influenza A (H1N1) is one of the most common virus strains causing influenza pandemics [2]. In March 2009, a pandemic outbreak, which began in Mexico, was identified as a new subtype of H1N1 [3]. Given its highly infectious nature, this new strain of influenza caused great concern globally, therefore the World Health Organization (WHO) raised its influenza pandemic threat level to six (the highest level) on 11 June 2009 [4] until 10 August 2010 [5]. After this period, the H1N1 influenza virus moved into a post-pandemic period. As of 1 August 2010, more than 214 countries and other territories or communities had reported confirmed laboratory cases of the 2009 H1N1 pandemic, including over 18,449 deaths [6]. 

In the early phase of this influenza pandemic, different countries faced different infection risks [7] and applied different non-pharmaceutical interventions against the 2009 H1N1 pandemic before the vaccine could be massively produced [8]. Many countries, including the United States and Canada, suggested that infected people should stay at home, and enforced school closures whenever necessary [9]. China adopted a much stricter prevention approach: one-week mandatory quarantine for all “close contactors” (*i.e.*, those who had close contact with an infectious person in hospitals or hotels) [10,11,12,13,14]. This prevention policy was carried out from the first identified imported (from the United States to China) case on 9 May [15] to 8 July 2009 [16]. On one hand, such strict preventative measures might cause misunderstanding over the justification of losing one’s movement freedom for a week, or even raise international conflicts (e.g., [17]). On the other hand, the effectiveness of quarantine for pandemic influenza is questionable, because the 2009 H1N1 pandemic was considered mild to moderate in seriousness; therefore, quarantines, as well as travel restrictions, were not recommended by the WHO [18]. Although hundreds of papers and reports have been published arguing for either side of the issue, no agreement has been reached concerning the cost effectiveness preparedness strategies and interventions against influenza pandemics [8].

It is a great challenge to fight effectively against a pandemic [19,20], especially when little is known about the new influenza [21], and historical influenza pandemics have not shown clear transmission patterns for reference [22]. In general, governments and medical services need rapid, rational, effective, and proportionate responses to such health emergencies; either minimalist or maximalist responses may potentially be very harmful [23,24]. However, dealing with a pandemic is a complex issue. The public health systems of some countries, e.g., Australia, were blamed for their failure to take steps necessary to respond to the 2009 H1N1 pandemic [25].

Whether to adopt strict prevention policies (e.g., mandatory quarantine) for an influenza pandemic or not is still an important question. As for the 2009 H1N1 pandemic, quantifying the effectiveness of interventions is one of the six challenges [20]. In this paper, we reviewed the transmission process of the 2009 H1N1 pandemic and the enforcement of quarantine in Beijing, China, and estimated the effectiveness of mandatory quarantine, in order to provide quantitative information for policy and decision makers, so as to develop more effective disease prevention strategies.

## 2. Methods

We used the Susceptible-Exposed-Infectious-Recovered (SEIR) model to estimate the dynamics of the 2009 H1N1 pandemic. China is a large country with heterogeneous local community conditions. The virus transmissibility varies in different regions. In order to provide a more realistic model, we only analyzed virus transmission and the effectiveness of quarantine in Beijing, China.

We compiled the data of daily confirmed cases of the 2009 H1N1 pandemic and number of quarantined individuals reported by Beijing Municipal Bureau of Health for the period when the mandatory quarantine was implemented (from 16 May to 8 July 2009). During the quarantine period all “close contactors” were isolated in hospitals or hotels for one week [10,11,12,13]. The definition of “close contactors” were published by the Ministry of Health of China [16]; for example, the close contactors in an aircraft are people that surround the infectious individual, with the maximum eight people (three in the front row, three in the back row, two on the left and right). In the meantime, any entrant at the customs with body temperature over 37.5 °C were also quarantined [16].

We simulated the pandemic dynamics using the estimated basic reproduction number *R_0_* in China [12,26], and evaluated the effectiveness of the mandatory quarantine.

### 2.1. The SEIR Model

The SEIR model has the form [27]:

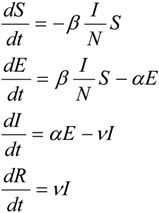

where *S* is the susceptible population, *E* is the exposed population, *I* is the infectious population, *R* is the recovered population, *t* is time (the number of days after the emergence of the first case), *N* is the total population, *β* is the average number of infected individuals per infectious subject per unit time, *α* is the reciprocal of average latent period, *v* is the rate of recovery (reciprocal of duration of the infection).

Until 31 December 2009, the number of accumulative confirmed cases (*i.e.*, 121,805 cases [28]) in mainland China corresponded to only 0.0094% of the total population. As such, we assumed the decreased percentage of susceptible population did not have significant effect on virus transmission in 2009. The seasonal pattern (waves) of the 2009 H1N1 pandemic was significant [28]. We assumed the infection rate decreased in the later stage of the transmission because of the season. We defined the decreasing infection rate linearly as below:

*β_t_* = *β* − (*t* − *t_peak_*) × *C*

where *t_peak_* is the number of days between the emergence of the first case and the peak of infection transmission, *β_t_* is the infect rate at the time *t*, *C* is a constant defining the decreasing rate of transmission.

### 2.2. Determination of Model Parameters

Fraser *et al*. [3] first calculated parameters of epidemiological transmission on the basis of the observations in Mexico from February to April, 2009, giving the basic reproduction number *R_0_* = 1.58, and average generation time *G* = 1.91 days. Based on laboratory-confirmed cases reported in Ontario, Canada, Tuite *et al*. gave the *R_0_* = 1.31, a mean latent period of 2.62 days, and a mean duration of infectiousness of 3.38 days [29]. For the transmission of the 2009 H1N1 pandemic in China, *R_0_* was estimated as 2.555 for the first peak and 1.886 for the second peak under control strategies [26]; and it was also estimated as 1.68 while school quarantine was implemented [12]. Based on the virus transmission records in Beijing [28], we calculated the *R_0_* value as 1.703, so that *β* = *R_0_*/G = 1.703/3.38 = 1.047 individual/day, using Tuite *et al*.’s result of duration of infectiousness [29].

Other model parameters are: total population size of Beijing, *N* = 20 million (based on the national population census in 2010); initial recovered population size, *R* = 4 million (*i.e.*, the initial proportion of the susceptible population was 80%); latent period (infected yet not infective), 1/*α* = 2.62 days [29]; rate of recovery, *ν* = 1/3.38 per day [29]. In Beijing, *t_peak_* = 2009/10/22 [28] – 2009/5/16 = 159 days.

We simulated infectious population of the 2009 H1N1 pandemic in Beijing from 16 May to 31 December, 2009 for the two scenarios: (1) nobody was quarantined; (2) close contactors were quarantined during the period from 16 May to 8 July, 2009. For the first and second scenarios, the virus transmission in Beijing started on 16 May and 8 July separately (see justification of the starting date in the Results section “Effectiveness of quarantine in Beijing” below). All other parameters for the two scenarios remain same.

## 3. Results

### 3.1. Summary of 2009 H1N1 Pandemic Infection and Control in Beijing and Mainland China

The first identified case of the 2009 H1N1 pandemic in mainland China was a passenger from the United States on 9 May 2009 [30]. Until 5 July, the number of accumulative confirmed cases was 1,040, including 758 cases that were imported from abroad and 282 local transmission cases [31]. Until 31 December 2009, 121,805 cases were reported in mainland China [28], while the number of imported cases is 2,254 [32]. The number of confirmed cases increased rapidly beginning at the end of August, and peaked by the end of November [28]. In Beijing, the first case (imported from the United States) was identified on 16 May 2009. On 5 July, the confirmed number of cases was 192, including 165 imported cases and 27 locally-transmitted cases [33]; the number of confirmed cases peaked by the end of October 2009 [28]. 

**Figure 1:**
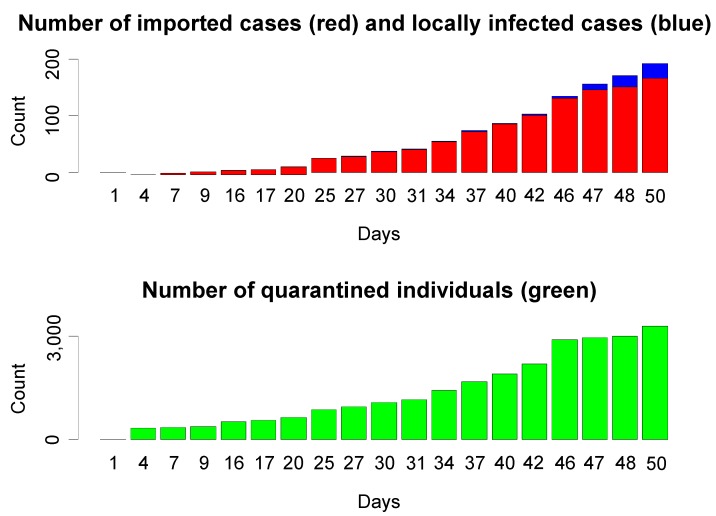
The cumulative number of imported cases and locally infected cases of the 2009 H1N1 pandemic, and quarantined individuals in Beijing during the mandatory quarantine enforced from 16 May (day 1) to 4 July 2009 (day 50).

From 10 May to 8 July, all “close contactors” who could be tracked down were quarantined for one week [16]. There is no official report of how many people in China were quarantined in total but a number of cities reported numbers of quarantined individuals. In Beijing from 16 May until 5 July, 2009, the accumulated number of quarantined individuals was 3,279 (Figure 1) [33].

### 3.2. Effectiveness of Quarantine in Beijing

In Beijing, there were 26 confirmed cases (all from abroad) and 865 quarantined “close contactors” from the first identified case on 16 May to the first presumably locally infection on 11 June 2009 (26 days later) (Figure 1). Until 30 June 2009, only three locally infected individuals were identified. On 1 July, infection was suspected in a primary school and seven cases among the students were confirmed later; the next day, ten more students and the staff of the school were also confirmed to be infected. From then on, locally infected cases increased rapidly. Since the effectiveness of quarantine was decreasing, the Ministry of Health announced the termination of mandatory quarantine on 8 July, 2009 [16].

**Figure 2:**
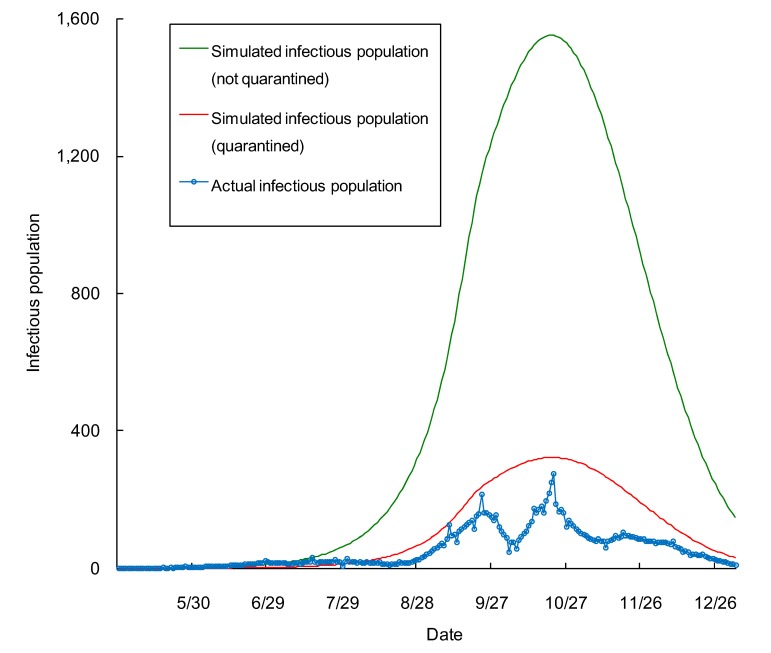
The simulated infectious population of the 2009 H1N1 pandemic in Beijing from 16 May to 31 December, 2009 for the two scenarios, quarantined (red line) and not quarantined (green line) (based on the SEIR model), and the confirmed number of infectious population (blue line).

We simulated the transmission of the H1N1 pandemic using the SEIR model (Figure 2). In the later stage (November and December 2009) the transmission rate became lower and the *R_0_* was adjusted (decreased 0.45%/day) to match the number of observed cases. We compared the sizes of the infectious population of the two scenarios (*i.e.*, virus transmission with or without mandatory quarantine). The only difference between the two scenarios was that the starting date of the quarantined scenario was postponed from 16 May to 8 July 2009; otherwise, all parameters for the SEIR model were same. We found that if no prevention measures were carried out, the infectious population would reach the maximum level at 1,553 individuals on 21 October, 5.6 times higher than the observed number (Figure 2). From May until the end of 2009, the intensity of the H1N1 pandemic measured by individual-day (infectious population times duration) was 26,622, based on the simulation using the SEIR model, when mandatory quarantine was taken into account. If no quarantine was implemented, the intensity would be 128,004 individual-days. The observed intensity was 13,305 individual-days, summed by daily infectious population (the missing values were estimated using the average values of adjacent dates).

The economic effectiveness of quarantine for the 2009 H1N1 pandemic is low. When a person was suspected to be a “close-contactor”, the person would receive several phone calls from different organizations (*i.e.*, the airline or railway company, local Center for Disease Control and Prevention (CDC), Ministry of Health, and local police station) to confirm the situation. Then, an ambulance with three to four CDC and medical service staff would come to pick up the person to a three star hotel. The hotel was exclusively used by those “close-contactors”, who would stay in the hotel for seven days. The cost of accommodations and medical checks was paid by government. We estimated the cost for scenarios with and without quarantine in Table 1. The scenario of quarantine cost 1.35 times higher than that of no quarantine.

**Table 1:** Cost effectiveness analysis for quarantine against the 2009 H1N1 pandemic in Beijing.

Scenario 1: nobody was quarantined				
	Quarantined individuals	Cost *per capita* (US$)	Cost (US$)	Memo
Tracking	3,279	120	3.93E+05	an ambulance with three staff
Accommodations	3,279	70 × 7 days	1.61E+06	seven days in a three star hotel
Medical check	3,279	50	1.64E+05	
	Infected individuals	Cost *per capita* (US$)	Cost (US$)	Memo
Medical check	26,622/3.38 * = 7876	50	3.94E+05	simulated intensity/duration
Total			2.56E+06	
**Scenarios 2: close contactors were quarantined during the period from 16 May to 8 July 2009**				
	**Infected individuals**	**Cost *per capita* (US$)**	**Cost (US$)**	**Memo**
Medical check	128,004/3.38 * = 37,871	50	1.89E+06	simulated intensity/duration
Total			1.89E+06	

* The duration of infectiousness is 3.38 days.

## 4. Discussion

Mortality and morbidity induced by the 2009 H1N1 pandemic was low [3]. Prevention measures should correspond to the severity and possible risk of the influenza pandemics [8,34]. Usually, a pandemic results in huge economic loss. In 2003, the SARS outbreak caused billions of direct losses and $30–100 billion of indirect losses [35]. The severity of influenza pandemic is lower than SARS, yet its size is much larger. Until now, researchers have conducted a number of quantitative analysis estimating of the economic loss of influenza. For example, every year regular influenza causes 5.6–8.5 billion pounds of economic loss due to lost working hours in France and Germany respectively [34]. In the United States, based on a conservative estimation, the cost of influenza to the US economy is about $167 billion annually [34]. However, the severity of the 2009 H1N1 pandemic was low, and the cost-benefit analysis is worth a special attention.

The overall economic effectiveness of mandatory quarantine that was enforced in Beijing was low. The peak of the 2009 H1N1 pandemic in Beijing would have been 5.6 times higher if mandatory quarantine had not been carried out in 2009. The mandatory quarantine had significantly delayed the peak of epidemic. However, the pool of susceptible population remains and in the longer run quarantine would not actually reduced the pandemic size. In Beijing, we estimated the direct cost (*i.e.*, tracking down close-contactors, accommodations and medical service) for quarantine, and the cost is higher than the cost of the scenario without mandatory quarantine (Table 1). The mandatory quarantine also has indirect cost, e.g., the loss of movement freedom and the interruption of the normal life of the people under quarantine, as well as people’s schedule changes due to the tense situation. The official budget for quarantine in mainland China is 96.6 million US dollars [14]. The indirect cost of mandatory quarantine would be much higher. For example, the indirect cost of SARS is ten times higher than the direst cost [35].

The transmission rate of the 2009 H1N1 pandemic was also low. Among 7,099 close contacts that were quarantined in China, the infection rate was 2.4% (167 of 7,099), ranging from 0.9% among aircraft passengers to >5% among household members [11]. That means over 95% quarantined people were unlikely to develop influenza symptoms. Other studies also show that the detection rate of border quarantine was 4.2% [14], and early interventions were not always effective [36].

Many mitigation methods are effective for a moderate influenza pandemic. The waiting for full pay policy in the workplace reduced the overall risk of an H1N1 pandemic by about 20% in one flu season in Japan [37]. Prevention policy of many countries shifted from containment to mitigation after sustained community transmission became established, such as in Singapore [38], South Korea [39] and the United Kingdom [40]. A systematic review suggests the reduction of nonessential contacts and the closure of schools, plus the use of pharmaceutical prophylaxis, are amongst the cost effective strategies for all countries; however, quarantine for household contacts is not cost effective even for low and middle income countries [8].

A number of studies performed a cost-benefit analysis of various prevention methods for the 2009 H1N1 pandemic, yet most economic evaluations focus on different interventions and compared to studies carried out before the 2009 pandemic [8]. We specifically analyzed the effectiveness of mandatory quarantine in Beijing during the transmission period in 2009, in order to deliver clear information for prevention of future influenza pandemics. We used the confirmed cases as the infectious population in the model. We need to note that a large proportion of cases (particularly asymptomatic and mild cases) would not have been detected. As such, the 2009 H1N1 pandemic was underestimated. Using the available data, we conclude that mandatory quarantine was not appropriate for the prevention of the 2009 H1N1 pandemic, which is a different conclusion than most other studies carried out in China (e.g., [12,26]).

## 5. Conclusions

The extensity of influenza pandemic prevention effort should correspond to its severity. After figuring out epidemiological parameters (e.g., transmission rate or basic reproduction number, probability of massive infection, and pandemic duration) and influenza severity (e.g., fatality and probability of developing complications), the decision makers should adopt appropriate intervention strategies. In 2009, China was in a post-SARS stage, and the government took a more radical strategy against the H1N1 pandemic for the first two months. Only after the recognition of massive local transmission did China terminate the mandatory quarantine policy. There is no quantitative system linking epidemiological parameters of a pandemic to intervention methods in China. To develop such a system would provide a solid foundation for science-based policy making.

## References

[1] Glasser J.W., Hupert N., McCauley M.M., Hatchett R. (2011). Modeling and public health emergency responses: Lessons from SARS. Epidemics.

[2] Layne S.P., Monto A.S., Taubenberger J.K. (2009). Pandemic influenza: An inconvenient mutation. Science.

[3] Fraser C., Donnelly C.A., Cauchemez S., Hanage W.P., van Kerkhove M.D., Hollingsworth T.D., Griffin J., Baggaley R.F., Jenkins H.E., Lyons E.J. (2009). Pandemic potential of a strain of influenza A (H1N1): Early findings. Science.

[4] WHO World now at the Start of 2009 Influenza Pandemic. Statement to the Press by WHO Director-General Dr Margaret Chan. http://www.who.int/mediacentre/news/statements/2009/h1n1_pandemic_phase6_20090611/en/index.html.

[5] WHO H1N1 in Post-Pandemic Period. http://www.who.int/mediacentre/news/statements/2010/h1n1_vpc_20100810/en/index.html.

[6] WHO Pandemic (H1N1) 2009—Update 112. http://www.who.int/csr/don/2010_08_06/en/index.html.

[7] Li X., Tian H., Lai D., Zhang Z. (2011). Validation of the gravity model in predicting the global spread of Influenza. Int. J. Env. Res. Public Health.

[8] Velasco R.P., Praditsitthikorn N., Wichmann K., Mohara A., Kotirum S., Tantivess S., Vallenas C., Harmanci H., Teerawattananon Y. (2012). Systematic review of economic evaluations of preparedness strategies and interventions against influenza pandemics. PloS One.

[9] Temte J.L. (2009). Basic rules of influenza: How to combat the H1N1 influenza (Swine Flu) virus. Am. Fam. Physician.

[10] Tang S., Xiao Y., Yuan L., Cheke R.A., Wu J. (2012). Campus quarantine (Fengxiao) for curbing emergent infectious diseases: Lessons from mitigating A/H1N1 in Xi’an, China. J. Theor. Biol..

[11] Pang X., Yang P., Li S., Zhang L., Tian L., Li Y., Liu B., Zhang Y., Liu B., Huang R. (2011). Pandemic (H1N1) 2009 among quarantined close contacts, Beijing, People’s Republic of China. Emerg. Infect. Dis..

[12] Tang S., Xiao Y., Yang Y., Zhou Y., Wu J., Ma Z. (2011). Community-based measures for mitigating the 2009 H1N1 pandemic in China. PloS One.

[13] Mei S., van de Vijver D., Xuan L., Zhu Y., Sloot P.M.A. (2010). Quantitatively evaluating interventions in the influenza a (H1N1) epidemic on china campus grounded on individual-based simulations. ProcediaComput. Sci..

[14] Liang W., Feng L., Xu C., Xiang N., Zhang Y., Shu Y., Wang H., Luo H., Yu H., Liang X. (2012). Response to the first wave of pandemic (H1N1) 2009: Experiences and lessons learnt from China. Public Health.

[15] Ministry of Health of China A Suspicious Case of Influenza A (H1N1) in Sichuan Province. http://www.gov.cn/gzdt/2009-05/10/content_1310043.htm.

[16] Ministry of Health of China Notification on Further Improving the Prevention and Control of Influenza A H1N1. http://www.gov.cn/gzdt/2009-07/08/content_1360236.htm.

[17] Castillo E.E., Ang A. Mexico Decries China’s Quarantine of Its Citizens. Huffington Post. http://www.huffingtonpost.com/huff-wires/20090504/as-china-mexico-swine-flu/.

[18] WHO Statement made at the Secretary-General’s briefing to the United Nations General Assembly on the H1N1 Influenza Situation. http://www.who.int/dg/speeches/2009/influenza_a_h1n1_situation_20090504/en/index.html.

[19] Shetty P. (2009). Preparation for a pandemic: Influenza A H1N1. Lancet Infect. Dis..

[20] Van Kerkhove M.D., Asikainen T., Becker N.G., Bjorge S., Desenclos J.C., dos Santos T., Fraser C., Leung G.M., Lipsitch M., Longini I.M. (2010). Studies needed to address public health challenges of the 2009 H1N1 influenza pandemic: Insights from modeling. PloS Med..

[21] Kamigaki T., Oshitani H. (2010). Influenza pandemic preparedness and severity assessment of pandemic (H1N1) 2009 in South-east Asia. Public Health.

[22] Hayden E.C. (2009). The turbulent history of the A(H1N1) virus. Nature.

[23] Tarantola D., Amon J., Zwi A., Gruskin S., Gostin L. (2009). H1N1, public health security, bioethics, and human rights. Lancet.

[24] Doyle A., Bonmarin I., Levy-Bruhl D., le Strat Y., Desenclos J.C. (2006). Influenza pandemic preparedness in France: Modelling the impact of interventions. J. Epidemiol. Commun. Health.

[25] Waterer G.W., Hui D.S., Jenkins C.R. (2010). Public health management of pandemic (H1N1) 2009 infection in Australia: A failure!. Respirology.

[26] Zhang J., Xiao Y. (2012). Modeling strategies for controlling H1N1 outbreaks in China. Int. J. Biomath..

[27] Hethcote H.W. (2000). The mathematics of infectious diseases. Siam Rev..

[28] Fang L.-Q., Wang L.-P., de Vlas S.J., Liang S., Tong S.-L., Li Y.-L., Li Y.-P., Qian Q., Yang H., Zhou M.-G. (2012). Distribution and risk factors of 2009 pandemic influenza A (H1N1) in mainland china. Am. J. Epidemiol..

[29] Tuite A.R., Greer A.L., Whelan M., Winter A.-L., Lee B., Yan P., Wu J., Moghadas S., Buckeridge D., Pourbohloul B. (2010). Estimated epidemiologic parameters and morbidity associated with pandemic H1N1 influenza. CMAJ Can. Med. Assoc. J..

[30] Center for Disease Control and Prevention (China) China Adjusts and Improves Prevention and Control Against Influenza A (H1N1). http://www.chinacdc.net.cn/n272442/n272530/n273736/n273781/n4624704/n4624713/32164.html.

[31] Simonov E.A., Dahmer T.D. (2008). Amur-Heilong River Basin Reader.

[32] Ministry of Health of China News Release Conference on Prevention and Control of Influenza A H1N1. http://www.china.com.cn/zhibo/2010-01/04/content_19174002.htm.

[33] Center for Disease Control and Prevention (Beijing) (2009). Status Report on Pandemic (H1N1) 2009 in Beijing.

[34] Szucs T.D., Nichol K., Meltzer M., Hak E., Chancelor J., Ammon C. (2006). Economic and social impact of epidemic and pandemic influenza. Vaccine.

[35] Keogh-Brown M.R., Smith R.D. (2008). The economic impact of SARS: How does the reality match the predictions?. Health Policy.

[36] Sato H., Nakada H., Yamaguchi R., Imoto S., Miyano S., Kami M. (2010). When should we intervene to control the 2009 influenza A(H1N1) pandemic?. Eur. Surveill..

[37] Miyaki K., Sakurazawa H., Mikurube H., Nishizaka M., Ando H., Song Y., Shimbo T. (2011). An effective quarantine measure reduced the total incidence of influenza a H1N1 in the workplace: Another way to control the H1N1 flu pandemic. J. Occup. Health.

[38] Tay J., Ng Y.F., Cutter J., James L. (2010). Influenza A (H1N1–2009) pandemic in Singapore—public health control measures implemented and lessons learnt. Ann. Acad. Med. Singap..

[39] Lee D.H., Shin S.S., Jun B.Y., Lee J.K. (2010). National level response to pandemic (H1N1) 2009. J. Prev. Med. Public Health.

[40] Balasegaram S., Glasswell A., Cleary V., Turbitt D., McCloskey B. (2011). From containment to community: Trigger points from the London pandemic (H1N1) 2009 influenza incident response. Public Health.

